# Thermal Imaging Metrology with a Smartphone Sensor

**DOI:** 10.3390/s18072169

**Published:** 2018-07-06

**Authors:** Leigh Russell Stanger, Thomas Charles Wilkes, Nicholas Andrew Boone, Andrew John Samuel McGonigle, Jon Raffe Willmott

**Affiliations:** 1 Department of Electronic and Electrical Engineering, The University of Sheffield, Portobello Centre, Pitt Street, Sheffield S14ET, UK; lrstanger1@sheffield.ac.uk (L.R.S.); naboone1@sheffield.ac.uk (N.A.B); 2 Department of Geography, The University of Sheffield, Winter Street, Sheffield S10 2TN, UK; tcwilkes1@sheffield.ac.uk (T.C.W); a.mcgonigle@sheffield.ac.uk (A.J.S.M.); 3 School of Geosciences, The University of Sydney, Sydney, NSW 2006, Australia; 4 INGV Sezione di Palermo, Via Ugo la Malfa, 153, 90146 Palermo PA, Italy

**Keywords:** thermography, metrology, quantitative thermography, infrared, thermometry, radiometry, thermal imaging

## Abstract

Thermal imaging cameras are expensive, particularly those designed for measuring high temperature objects with low measurement uncertainty. A wide range of research and industrial applications would benefit from lower cost temperature imaging sensors with improved metrology. To address this problem, we present the first ever quantification methodology for the temperature measurement performance of an ultra-low cost thermal imaging system based on a smartphone sensor. The camera was formed from a back illuminated silicon Complementary Metal Oxide Semiconductor (CMOS) sensor, developed for the smartphone camera market. It was packaged for use with a Raspberry Pi computer. We designed and fitted a custom-made triplet lens assembly. The system performance was characterised with a range of state-of-the-art techniques and metrics: establishing a temperature resolution of below 10 °C in the range 600–1000 °C. Furthermore, the scene dependent aspects of combined uncertainty were considered. The minimum angular subtense for which an accurate thermal measurement could be made was determined to be 1.35°, which corresponds to a 23 mm bar at a distance of 1 m, or 45:1 field-of-view in radiation thermometer nomenclature.

## 1. Introduction

Thermal imaging, thermography and radiation thermometry are widely applied techniques within a variety of manufacturing industries, military applications, medical diagnostics and academic research domains. The most common commercial applications of thermography are found in semiconductor processing [1,2,3,4], the plastics industry [5,6] and metals processing [7,8,9,10]. In addition to these applications that are significantly above ambient temperature, thermography under ambient conditions is also applied within the medical sector [11,12].

Thermography involves the capture of images, which resolve the distribution of radiant exitance across a scene [13]. The most common implementation of thermography is achieved by measuring the infrared radiant flux that is captured by the pixels of Focal Plane Arrays (FPAs); this is a radiometric measurement. Although often assumed to be quantitative, in a strict metrological sense qualitative imaging is most common, with the design effort being focused upon Minimum Resolvable Temperature Differences (MRTDs), which constitutes a subjective measure of performance that was initially pioneered for military applications [14,15]. An alternative, objective measure of temperature resolution is Noise Equivalent Temperature Difference (NETD) [13,16]. Quantitative thermal imaging requires an assessment of the individual components of uncertainty [8], which contribute to the overall error in measured temperatures [17].

Radiation thermometry is a highly developed metrological domain aimed at establishing quantitative temperature measurements with low uncertainties. The International Temperature Scale of 1990 (ITS-90) [18] defines the traceability to the Kelvin definition. Traceability is realised by the use of highly linear single pixel Infrared Radiation Thermometer (IRT) measurements above the freezing point of silver (961.78 °C) and the resistivity of high purity platinum at all lower temperatures.

Metrological IRTs have well-characterised sensor responses to incident radiance and minimal internal stray radiation. However, these units are also expensive and delicate, with complex optical systems, hence they are impractical for industrial applications. In contrast, commercial IRTs have less well-characterised sensors, simplified optical systems and less well-defined Measurement Fields Of View (MFOV). MFOV may be expressed as the ratio of object distance to measurement area, as an angular subtense, or a measurement diameter at a fixed working distance.

The degree of stray light within an IRT is characterised by the Size of Source Effect (SSE) performance metric [19,20], which defines how sensitive the thermometer is to the radiance of the scene surrounding the intended measurement area. Thermal imaging cameras tend to have very poor SSE [21], which is in contrast to high quality radiation thermometers. Thermal imaging cameras with well-designed optics and isolated pixels in the FPA have the lowest SSE. SSE is normally characterised by a Point Spread Function (PSF) [22] or Modulation Transfer Function (MTF), which is the Fourier transform of the PSF [19]. Low cost quantitative thermal imaging requires correction for, or assessment of, the uncertainty introduced by the comparatively poor SSE.

With the rapid escalation in smartphone usage over the last decade, compact, low cost optical sensors have become readily available. These are based on the silicon Charge Coupled Device (CCD) and, more recently, Complementary Metal Oxide Semiconductor (CMOS) design format. By combining such devices with recently developed inexpensive, distributable and networked computer boards (e.g., Raspberry Pis) the way is now open to consider the potential of this technology in remote quantitative temperature measurement applications. Indeed, we anticipate that such devices could form the basis of distributed thermal monitoring networks in industry or very low cost standalone portable thermal camera units. In this article, we explore the novel metrological possibilities by reporting the first quantification of the temperature measurement performance of an ultra-low cost thermal imaging camera based on a smartphone sensor. This work is focused on quantitative thermography with a Near InfraRed (NIR), FPA system, based on a (£ 20) Raspberry Pi camera, which contains a sensor developed for the smartphone market.

In particular, we report a metrologically rigorous thermographic calibration and assessment protocols, which were applied to establish the system performance. The intention of this work is to expedite the uptake of quantitative thermal measurements using low cost off-the-shelf consumer electronics. Given the very low cost of the Raspberry Pi cameras (PiCams) applied in this study, in contrast to the typically >£ 10k price point of the Si CMOS based units conventionally applied in this arena, our work has the potential to significantly broaden the current reach of thermal metrology.

This research builds upon a number of other recent reports on novel applications of these low cost array sensors [23,24,25,26] and the assessment protocols presented here could be applied to any NIR FPA based temperature imaging system above temperatures of around 500 °C. Throughout this article, for adopted protocols and associated vocabulary, we follow the two relevant ISO standards relating to thermography in the context of Non-Destructive Testing [27,28].

## 2. Theoretical Basis for Quantitative Thermography

The main factors which determine the uncertainty budgets of thermographs are: the calibration of the detector response to incident radiance; the intrinsic noise in the system; the spatial transfer function of the device; and the emissivity of, and reflected radiation from, the elements within the scene.

Uncorrelated components of uncertainty can be combined to establish overall error via quadrature addition [29]:

(1) $$U_{y}^{2}\;=\;\underset{i}{\sum }{\left(\frac{\partial y}{\partial x_{i}}\cdot U_{x_{i}}\right)}^{2}.$$

In this case $U_{y}$ is the combined standard uncertainty in the measurand y (surface temperature), $x_{i}$ is the *i*th parameter which contributes to the total uncertainty and $U_{x_{i}}$ is the standard uncertainty in this parameter. A measurement model characterising the dependence of the measurand on all the relevant parameters $y\left(\left\{x_{i}\right\}\right)$ is also required. In scenarios where such a model cannot be constructed, the total measurement uncertainty can be established instead via Monte Carlo methods [30].

### 2.1. Radiometric Calibration

The treatment applied here is predicated for blackbody radiators, the spectral radiance from which follows Plank’s Law. Radiometric calibration is based on measurements performed at stable discrete temperatures, whereby a functional form of the response of the detector is used to interpolate between calibration points. Currently, the most popular interpolation approach for narrow band radiometers is the Sakuma Hattori equation [31], which defines the expected measured signal $S_{meas}$ of any individual pixel with a uniform temperature MFOV, to be:

(2) $$S_{\mathrm{meas}}\left(T\right)\;\approx \;S_{model}\left(T\right)\;=\;\varepsilon^{*}\beta S_{S-H}\left(T\right),$$

this assumes that the surface is freely radiating, and is based on: $\varepsilon^{*}$, the effective emissivity of the measurement; β, the transmission coefficient between the surface and the thermographic device; and $S_{S-H}$, the Sakuma Hattori model for a narrow band IRT response to blackbody radiation at a known temperature T(K), such that:

(3) $$S_{S-H}\left(T\right)\;=\;A_{0}/\exp\left(\frac{c_{2}}{A_{1}T+A_{2}}\right)-1.$$

Here $c_{2}$ is the second radiation constant, 1.43877736(83) × 10^−2^ (m K) [32] and $A_{0}$, $A_{1}$ and $A_{2}$ are parameters which can be fitted to calibration data or calculated with knowledge of the spectral system response function [33]. Calibration is carried out by overfilling the sensor element MFOV with spatially uniform quasi-blackbody radiation.

### 2.2. Spatial Transfer Function

The fidelity with which a measurement system can resolve the physical phenomenon under observation is characterised by the device transfer function. In optical imaging systems, the spatial deformation of the scene associated with formation of an image is described by the Optical Transfer Function (OTF). The OTF can be expressed as a complex function of spatial frequency and phase, such that the modulus of the phase independent part of the OTF is the MTF. In spatial frequency space the resultant image is the product of the scene and the OTF of the system. If we consider the OTF to be position and orientation independent, then this function will contain only real components and, therefore, be fully described by the MTF; as is assumed to be the case in most thermographic literature [17,19,20,22]. Further distortion to the digital scene representation is introduced by the sensor itself; such that the final measured MTF of a captured digital image is affected by the optics, electronics and digital data manipulation.

The measurement area or MFOV [28] of a single pixel IRT is typically defined as the angular subtense from which 95% of the radiation contributing to the IRT reading originates. The radial distribution of contributions from the scene to the pixel measurement is characterised by the SSE and MFOV. The area at which the pixel is imaged directly onto the scene is termed the Instantaneous Field of View (IFOV) [28]. Hence, the radiant flux incident upon each pixel is influenced by multiple surrounding IFOVs, and, for pixels close to the edge of the image, by the scene outside the FOV of the sensor array.

The contribution of surrounding IFOVs to the MFOV of each pixel can be characterised by a PSF, which is typically defined over a small number of pixels, e.g., a 15 × 15 array used in a recent report by NIST [8]. In this case the PSF was modelled as a radially symmetric exponential decay function. However, effects from rather further afield can be significant too [21]. It has been proposed that de-convolution of IR device images with the unit PSF function [8,9] will produce a more accurate representation of the temperature distribution across the scene. In this case, uncertainty in measurement of the PSF has been shown to be the dominant source of uncertainty in final measured temperatures when utilising this deconvolution process [8].

Many long wavelength FPA devices have significant inhomogeneities in their responsivities to radiation across the sensor array, a phenomenon known as fixed pattern noise. However, the PiCam sensors used in this study have a relatively uniform response across the field. In principle, inhomogeneity can be digitally corrected, by capturing a map of ‘dead pixels’. Furthermore, a uniform radiance scene, which fills the entire sensor FOV, can be used to correct the non-uniformity of the responsivity of each pixel to a flat field. However, this was not deemed necessary for the PiCam device and, instead, the minor deviation from a perfect flat field was included as a component in the final uncertainty budget.

### 2.3. Performance Characterising Metrics

We have defined the temperature measurement resolution to be the spatial frequency at which the MTF metric is 0.95. This is analogous to the definition of the MFOV of an IRT which is: the ratio of the diameter of the overall measurement area to the in-focus working distance. The overall measurement area is calculated as a percentage (typically 95%) of the total radiance [28]. We have, therefore, defined spatial variations below this spatial frequency to contain accurate radiometric measurements. Furthermore, the imaging resolution can be defined as the spatial frequency corresponding to an MTF of 0.5, such that image areas with spatial frequencies lower than this value are taken to have sufficient resolution for imaging purposes. Both of these criteria accord with standard practices.

The MRTD is a commonly used performance metric defined as the ‘measure of the ability of an infrared imaging system and the human observer to recognise periodic bar targets on a display’ [28]. MRTD is, therefore, a subjective metric that is dependent upon the user and the display modality of the system. An alternative objective performance measure is the temperature resolution or NETD, defined as the ‘target-to-background temperature difference between a blackbody target and its blackbody background at which the signal-to-noise ratio is equal to unity’. This can be directly measured by observing a scene with two narrowly spatially separated blackbody temperatures $T_{1}$ and $T_{2}$, with corresponding measured signals of *S*_1_ and *S*_2_. The NETD is described by Minkina and Dudzik [16] as:

(4) $$\mathrm{NETD}\;=\;\frac{\sigma_{s}}{\left(S_{2}-S_{1}\right)/\left(T_{2}-T_{1}\right)},$$

where $\sigma_{s}$ is the root mean square, or standard deviation noise, in the signal measured at *T*_1_ or *T*_2_. Using the modelled signal (2), the derivative of the Sakuma Hattori model, with respect to temperature, can be applied with the noise measured at a single blackbody temperature to provide an experimentally accessible measure of NETD:

(5) $$\mathrm{NETD}\;=\;\sigma_{s}\cdot {\frac{\partial S_{meas}}{\partial T}}^{-1}.$$

### 2.4. Quantitative Thermography

An accurate surface temperature can be ascertained utilising this device, if due care is taken when assessing the scene. The quantities $\varepsilon^{*}\;\mathrm{and}\;\beta$ must be known and assumed constant across the MFOV. The surface temperature must be constant across the MFOV. A dark image correction is applied to the signal level from each pixel, to account for any drift in the device, for example, from changes in ambient temperature. With these steps taken, isolated objects can be accurately measured. Further corrections are required where there are multiple sources of infrared radiation across the scene. Contributions such as reflected radiation, to the radiance from the object to be measured, must be properly considered and corrected for by subtracting the deduced additional signal from the other objects. Once these factors are taken into account Equation (2) is inverted to give the surface temperature in Kelvin for each pixel.

## 3. Experimental Procedure

The sensor used in our thermal imaging system design was a modified Raspberry Pi Version 1.3 camera. The camera’s Bayer filter was removed [23] to allow the longest wavelength, NIR sensitivity range of the sensor to be utilized and a Schott RG850 coloured glass long pass filter was added to the optical system. This procedure also removed the mosaic pattern response across the FPA. The main properties of the sensor, including the custom fore optical configuration described below, are presented in Table 1. During image acquisitions, the exposure time was set to 1.5 ms and analogue gain to 1, which set the temperature range over which the instrument measured.

The blackbody radiator we used as the characterised laboratory radiance source was a ‘Landcal P1200B’ unit, with a 300 mm long, 50 mm diameter, cylindrical cavity; which was open at one end and terminated at the other. The terminal end was formed from a 120° angle cone. The cavity was machined from cast silicon carbide with a surface emissivity of ~0.9, according to the manufacturer supplied documentation. The ε^*^ of the cavity, enhanced by multiple internal reflections, is quoted at 0.998 for isothermal conditions. A calibrated R-type reference thermocouple, inserted into a hole machined in the cavity body, was used to derive reference blackbody temperatures. This also enabled traceability of the instrument calibration to the ITS-90 standard through prior calibration of the thermocouple in a UKAS accredited laboratory. Temperature gradients along the length of the cavity will move the furnace away from blackbody conditions. 

A 500 mm long Inconell sheathed, type-K thermocouple was used to measure the temperature gradients along the cavity by moving it to different locations inside a channel in the furnace that ran parallel with the top of the blackbody cavity. An orifice was integrated into the design of the furnace, for this purpose. A + 10 °C offset to the right-hand zone of the furnace was found to minimise these gradients over the temperature range of interest. Temperature gradients along the blackbody cavity wall may also cause the cavity ε^*^ to become spectrally dependent [34]. An estimate of the uncertainty in ε^*^ introduced by these minimised cavity spatial inhomogeneities was made by calculating the angular field of view occupied by the different temperatures and comparing this to the perfectly uniform ε^*^ scenario quoted in the manufacturer’s documentation. This was established to be 4% (k = 2) over the range of temperatures applied in our experiments.

### 3.1. Optics

**Figure 1:**
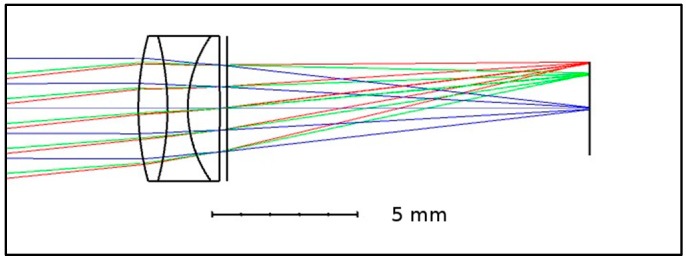
Ray trace diagram of the custom designed triplet lens from our thermal imaging camera design. The lens was designed using the commercially available ray tracing software, Optic Studio. All the optical elements were stock items.

The optics affixed to the fore of the sensor consisted of a triplet made using three off-the-shelf optical elements (see Figure 1): a Ross Optical, L-BCX011 Effective Focal Length (EFL) = 18 mm lens; a Thorlabs, LD4797 EFL = −6 mm lens; and an OptoSigma, SLSQ-07B-08P EFL = 8.7 mm element. These were mounted in a plastic 3D printed enclosure, which also formed the mount for the sensor board and removable optical filter (RG850, Thorlabs, Ely, UK).

### 3.2. Radiometric Spectral Responsivity of the Filtered Sensor

**Figure 2:**
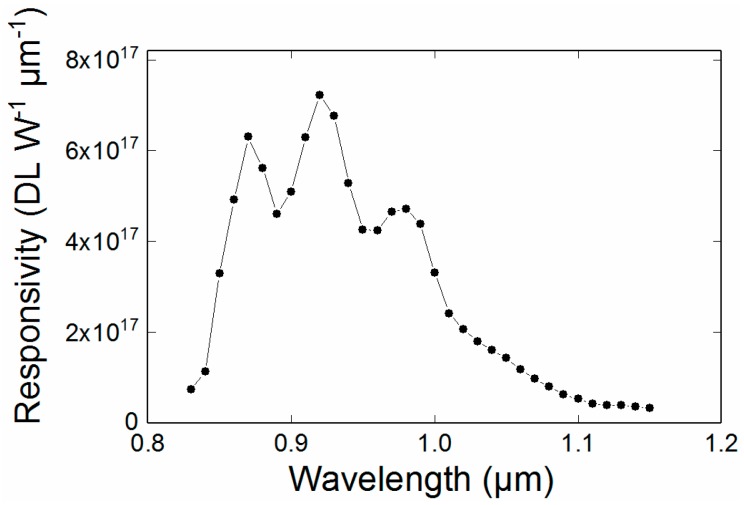
Spectral responsivity of the modified PiCam combined with an 850 nm long pass filter. The oscillatory behaviour is consistent with being caused by etalons in the Bayer removal process. This spectral shape affects the form of the calibration curve as a function of temperature.

A monochromator was used to measure the overall spectral responsivity of the system; the indicative results are shown in Figure 2. The monochromator generated spectrally adjustable narrow band light of approximately 2 nm full width at half maximum. The absolute intensity of the light was not critical because it was removed as a scaling factor within the calibration, $A_{0}$ in (2). In this case the relative spectral intensities emitted by the source were measured using a characterised commercial InGaAs detector to correct for the spectrally non-uniform emission spectrum of this device. Figure 2 shows that the responsivity spectrum was broadly similar to the expected, typical silicon detector response, once the Bayer filter was removed. The measurement of the spectral sensitivity forms a starting point for the fitting of the Sakuma-Hattori Equation, but does not affect the outcome of the final calibration and is included for visualisation purposes only.

### 3.3. Radiometric Calibration

A 25 mm diameter circular aluminium aperture was placed close (~10 mm) to the front aperture of the furnace, to reduce scatter and provide a uniform target. This aperture did not exceed 100 °C in temperature, which was well below the camera detection limit. The optical axis of the camera system was aligned parallel to that of the blackbody cavity, with the centre of the sensor located on the central axis of the cavity. This minimised the inhomogeneity otherwise caused by viewing of the cavity walls. The separation between the front surface of the lens and the furnace aperture was (450 ± 5) mm. The optics were adjusted to bring the aperture into precise focus. The three-zone furnace had a fixed +10 °C offset on the front zone in order to minimise thermal gradients along the cavity. Dark images were captured by periodically, at least once daily, by covering the PiCam with a lens cap. The digital levels of the dark image drifted insignificantly over both short (a period of hours) and long (a period of days) timescales. We found that the ambient temperature in the laboratory did not deviate from the range 22–28 °C, which was measured by a calibrated platinum resistance thermometer. The PiCam is not temperature controlled the dark image levels may drift significantly when exposed to extreme ambient temperatures, therefore, dark images should be captured and subtracted with every use of the camera. Dark images were subtracted from all captured images. Each calibration point was recorded as the mean of the pixel values over the central measurement Region of Interest (ROI). This consisted of 12.5 × 10^3^ pixels, imaged at the middle of the furnace aperture. We verified experimentally that the ROI measurement was unaffected by changes in radiance due to different silhouetting features (masks) we placed in front of the furnace aperture, where the diameter of the aperture was chosen to be twice the diameter of the MFOV. In order to investigate noise statistics in these ROI calibration data, frame to frame average and standard deviations were determined 30 sequentially captured images. A representative image of the calibration furnace is shown in the Results and Discussion section.

**Figure 3:**
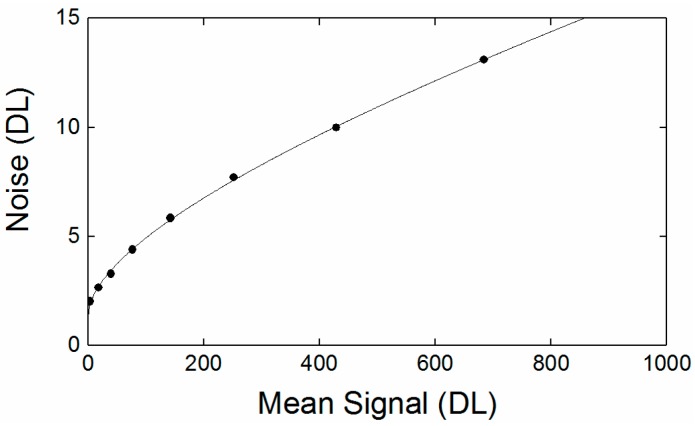
Digital level (DL) mean temporal noise levels plotted against signal, showing a near square root dependence, suggesting that the system is quasi-shot noise limited.

The standard deviation derived noise as a function of signal strength is shown in Figure 3, displaying square root dependence, which implies that the system was largely shot noise limited. 

### 3.4. Spatial Transfer Function Measurement

**Figure 4:**
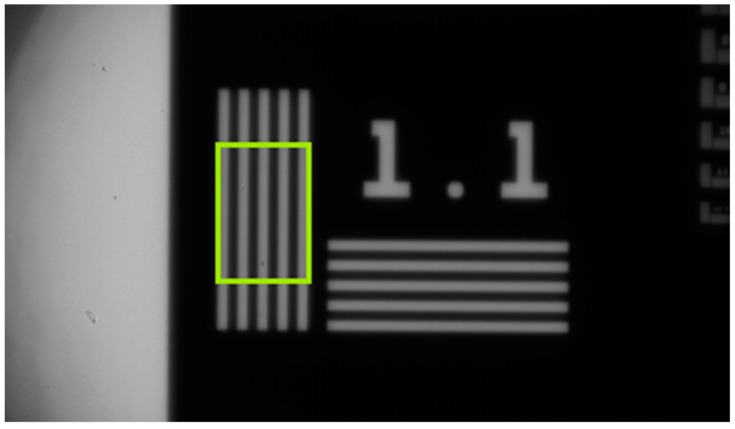
A representative image of the commercial NBS1963A CTF plate showing the ROI used to extract the CTF values, which were then converted to an MTF.

The spatial transfer function was measured with a commercially available NBS 1963 Contrast Transfer Function (CTF) target for spatial frequencies (f) between 1 line pair per mm (lp mm^−1^) and 5 lp mm^−1^. It was also necessary to use a custom-made laser cut metal target with spatial frequencies between 1/14 lp mm^−1^ and 1 lp mm^−1^. The NBS plate consisted of a chromium pattern mounted on the surface of a soda lime glass slide, which had a transmission measured to be 92.4% over the band pass spectrum of the system. This was measured by assuming the radiance to be constant at the cross over point between the two targets (1 lp mm^−1^) and taking the ratio of the measured modulation amplitudes. The custom-made target was composed simply of lines cut in a plate and, therefore, had transmission of 100%. Test plates were placed in front of the furnace aperture so that they could be imaged by our camera, as shown in Figure 4. The plates were imaged 30 times, for each test, and the mean values recorded. The resulting images were analysed using a rectangular ROI placed over the bars sufficiently far from the bar ends to avoid edge effects (Figure 4). The intensity profile was measured perpendicular to the bars by taking the maximum ($S_{max}$) and minimum ($S_{min}$) intensity of the central phase of the target:

(6) $$\mathrm{CTF}\left(f\right)\;=\;\frac{\left(S_{max}-S_{min}\right)}{\left(S_{max}+S_{min}\right)},$$

whilst MTF measurements require sinusoidal inputs, the CTF plates consisted of bar targets, resulting in square wave functions. The measured CTFs were converted to MTFs by considering the higher order components of the CTF and correcting for them using the Coltman formula [35]:

(7) $$\mathrm{MTF}\left(f\right)\;=\;\frac{\pi }{4}\left[CTF\left(f\right)+\frac{CTF\left(3f\right)}{3}-\frac{CTF\left(5f\right)}{5}+\ldots \right].$$

### 3.5. Flat Field

A gold-coated collimating mirror was used to fill the camera FOV with illumination from the blackbody cavity at 1000 °C. An exposure time of 1 ms gave a mean signal of 613.6 Digital Levels (DLs). The standard deviation of pixel values across the entire camera FOV, after the ‘dead’ pixels were removed with a mask, was found to be 17.7 DL. The ratio 0.03 was used as an estimate of the statistical distribution of pixel levels across the FOV.

## 4. Results and Discussion

**Figure 5:**
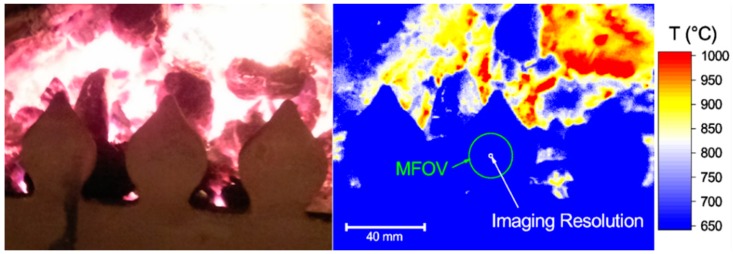
A representative thermal image of a lit domestic wood and coal burning stove. The imaging resolution is sufficient to resolve details of the scene. The MFOV determines the minimum uniform feature size for which a truly quantitative measure of the temperature can be made.

A thermal image of a domestic wood and coal burning stove is shown in Figure 5, alongside a contemporaneous visible wavelength photograph. The imaging resolution of the PiCam thermal camera allowed thermal features of the scene to be clearly observed and temperature measurements to be made over the varying thermal distributions. Compared to equivalent thermal imaging cameras, Figure 5 represents very high resolution imaging with well characterised measurement uncertainty.

**Figure 6:**
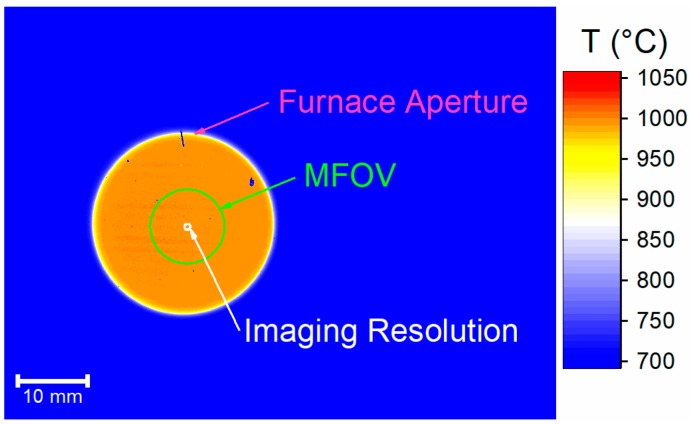
A representative calibration image of the furnace aperture with the furnace at 1000 °C. The three circles respectively show: furnace aperture area, minimum area over which an accurate temperature measurement can be made, and area over which thermal distributions can be resolved.

Figure 6 is a representative thermal image of the calibration furnace, showing the uniform radiance region, illustrating that for a uniform target of a similar size, our thermal imaging camera produces a truly quantitative measure of radiance temperature. The labelled circles show the different spatial resolutions for our camera. These circles are reproduced in Figure 5, allowing determination of the spatial region (‘size’) over which an accurate temperature measurement can be made and the spatial region over which different thermal gradients can be resolved. The former is analogous to the requirement for a radiation thermometer that an object must fill the instrument FOV if an accurate measurement is to be made.

**Figure 7:**
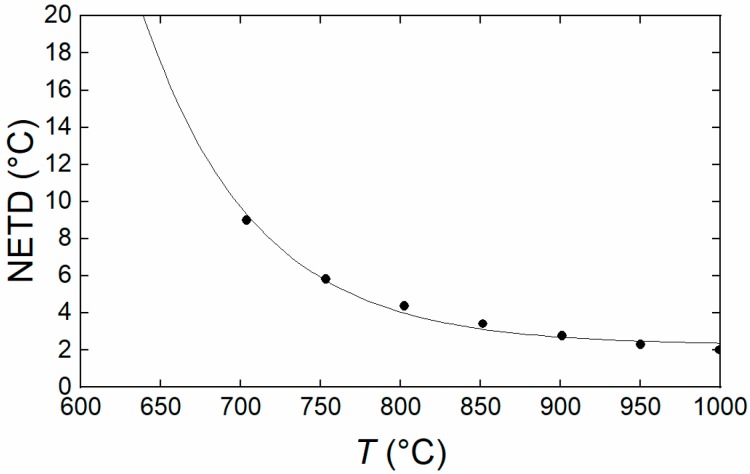
The Noise Equivalent Temperature Difference for the Thermal PiCam system. Each value is taken as the mean across the entire image. The NETD can be reduced by √N with pixel averaging where N is the number of pixels in the mean average.

Temperature resolution data for the PiCam are shown in Figure 7. The resulting instrument performance is comparable to that of commercially available Si thermographic devices such as the LumaSense Mikron MCS640 instrument [36]; which has a quoted resolution of 1 °C at 600 °C with a MFOV of 0.9 mrad (using their standard lens). The quoted uncertainty for the Mikron MCS640 is 0.5% of T (K), which can be contrasted with that of the AMETEK Land Cyclops100 L IRT [37], which has quoted metrics of 0.25% of T (K) and 180:1 FOV. Hence, our Thermal PiCam thermal imaging camera has appropriate performance metrics for the many industrial and research applications that the aforementioned commercial instruments are used for, with the significant benefit of considerably reduced price and superior pixel count. The combined standard uncertainty of our camera dependent components of the final measurement uncertainty are shown in Table 2. A representative calculation of the combined uncertainty yields a quantitative temperature measure of temperature of (800.0 ± 5.4) °C (*k* = 1), with an assumed $\varepsilon^{*}$ of one with zero uncertainty and no averaging of the pixels. Averaging of 4 pixels yields a reduced measurement uncertainty of 3.7 °C at 800.0 °C. The thermocouple calibration uncertainty was ascertained directly form the calibration certificate provided by the manufacturer. The back-wall discrepancy was estimated from the same spatial profiling technique used to estimate the temperature inhomogeneities along the length of the cavity. This was the maximum expected difference in temperature between the position of the reference thermocouple and the back wall of the cavity. The blackbody radiator $\varepsilon^{*}$ was calculated using the method described in Section 3. The standard error of estimate was the standard deviation of the residuals of the fit of the measurement data to the Sakuma-Hattori measurement model [8].

The non-flat-field was the fractional uncertainty in the signal introduced by the difference in signal levels for a uniform radiance object throughout the scene. This was estimated using a collimating mirror to fill the FOV of the instrument. The noise was the average image to image variations in each pixel when observing a constant radiance object. The noise was dominated by the shot noise of the object being measured. As indicated by the square root dependence of the noise on signal level shown in Figure 3.

**Figure 8:**
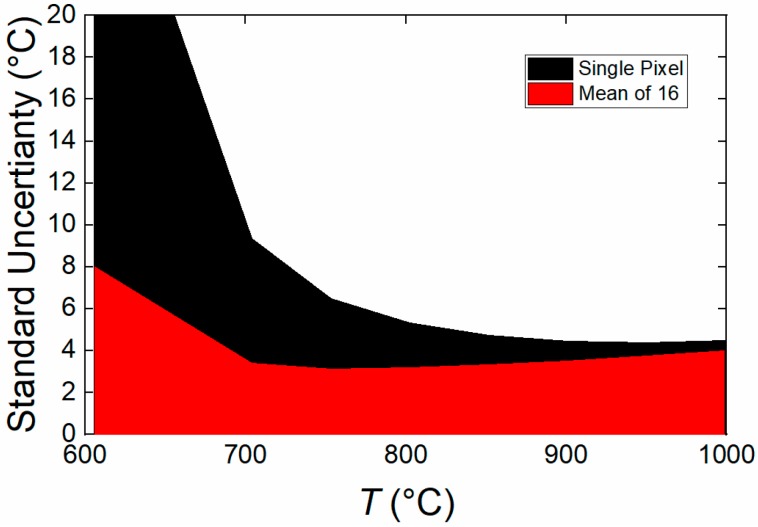
The combined Standard Uncertainty (*k* = 1) of the Thermal PiCam when used as a thermal camera in the 600–1000 °C range. The averaging of pixels significantly reduces measurement uncertainty at the lower temperatures.

Figure 8 shows the benefits of averaging the pixel values either temporally, or spatially, to reduce the random noise in the measurements from our camera, which is its dominant source of measurement uncertainty.

**Figure 9:**
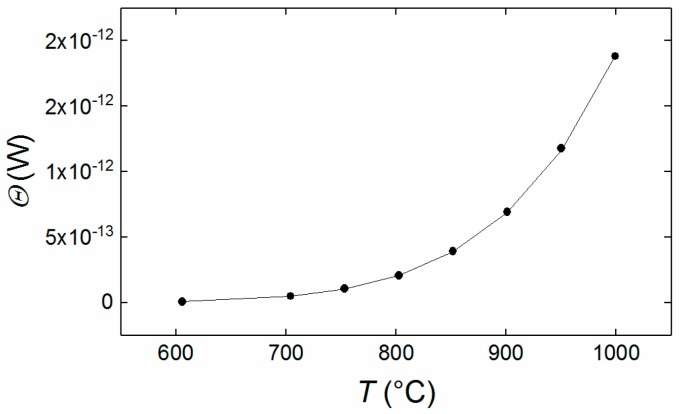
Calibration curve for 1.5 ms exposure time showing an exponential dependency of irradiant flux (*Θ*) upon temperature at the operating wavelength. The points are measured values at an assumed spatial frequency of zero and the line is the best fit of the Sakuma Hattori calibration curve with an assumed ε^*^ value of 0.998.

**Figure 10:**
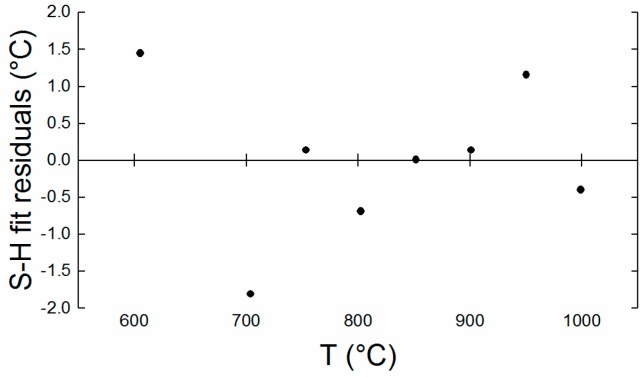
Residuals of the Sakuma-Hattori fit. These values are significantly smaller than the uncertainty of each of the measurement points. The standard error for these data is 1.03 °C

The irradiant flux values (*Θ*) presented in Figure 9, were calculated using the optical transmission factors derived from the ray tracing model of the optical system. This was combined with the radiometric calibration, to determine the integrated responsivity characteristics of the camera system, as shown in Table 3. The residuals of the Sakuma-Hattori fit are shown in Figure 10; these are small and random, implying that the calibration fit is very good.

**Figure 11:**
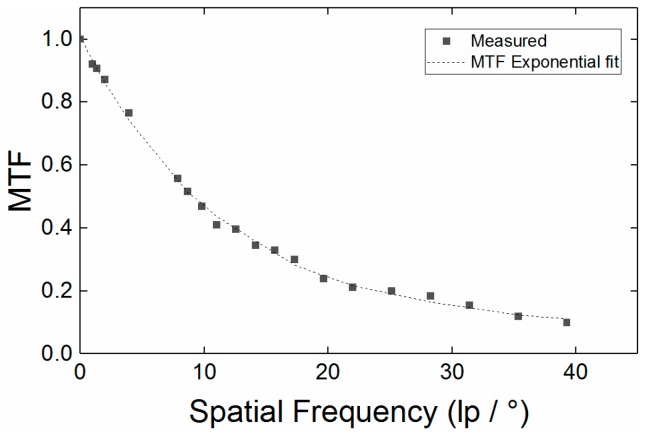
Plot of the modulation transfer function vs. spatial frequency. Following our definition of FOV by analogy to radiation thermometry, the minimum spatial frequency for a temperature measurement at which MTF is ≥95% is 0.74 lp mm^−1^. This corresponds to an object approximately 10 mm wide at a working distance of 450 mm. Our temperature MFOV is, therefore, 45:1. An exponential decay with an offset was used to fit these data.

The MTF curve in Figure 11 shows the response of our Thermal PiCam sensor based camera to high spatial frequencies. The largest contribution to the reduction modulation with frequency is assumed to be pixel cross talk within the camera sensor. This observation is corollary to the optical raytracing that determined the optical component of MTF to be unity over the whole range of frequencies in Figure 11.

Overall, our results demonstrate that the inexpensive PiCam CMOS sensor can be used for quantitative thermal imaging with comparable performance to commercial cameras that are up to two orders of magnitude more expensive. This opens up thermal imaging to high temperature research and manufacturing fields, in a modular, reconfigurable format that could compliment or replace single point radiation thermometry in many applications. In particular, the Pi’s communication features, could enable distributed thermal imaging with several spatially separated instruments. Sensors like this could form sensor systems within factories of the future and industry 4.0 applications, for mapping temperatures above 500 °C.

## 5. Concluding Remarks

In an attempt to meet the need for inexpensive quantitative thermal imaging for industrial and research applications, we have reported the first quantitative assessment of the performance of a smartphone sensor-based thermal imaging camera for quantitative thermographic applications. In particular, we have built a very low cost unit consisting of a Raspberry Pi camera, electronics, sensor and lens system, at a cost at least an order of magnitude lower than commercial units which are currently deployed to this end. In particular, the tests detailed in this paper demonstrate our camera’s temperature resolution to be better than 10 °C, in the range 700 °C to 1000 °C, with an angular MFOV of 23.6 mrad. By analogy to radiation thermometry, we find that our camera measures temperature with a FOV of 45:1. We find the PiCam based system to be closely comparable in performance to a commercial FPA-based thermographic instrument, which retails at a price point of around £ 15k. In addition to price point, a further advantage of our Thermal PiCam is the factor of 16 greater number of pixels. This increased pixel count could be used for improved discrimination of thermal distributions or to reduce electronic noise by means of co-adding pixel measurements.

By analogy to radiation thermometry, we have developed new thermal imaging camera characterisation methodologies. These allow, for the first time, radiation thermometer performance metrics to be compared with thermal imaging camera performance. The well characterised MFOV of our camera provides confidence in its capacity to deliver quantitative measurements of temperature within the aforementioned range of temperatures and spatial frequencies. We have characterised the different extents of our thermal images over which thermal distributions can be qualitatively mapped and temperature measurements can be quantitatively measured. This coupling of a low cost quantitative thermographic device with the readily networkable Raspberry Pi computer platform provides significant potential in industry 4.0 applications, most obviously where high temperature processes requires monitoring and control based feedback. Furthermore, the processing power of the Raspberry Pi camera permits front-end data reduction, allowing the camera to operate as a smart sensor. The device also provides an easily accessible, low cost research tool, benefitting from the existing wide reach of the native open source Python coding platform, which could expedite uptake in the widest possible sense e.g., amongst the maker community and as a teaching tool in schools, further and higher education. Whilst the measurements uncertainties detailed here appear modest relative to the highest quality radiation thermometers, this device is of utility in quantitative thermographic fields, these values may be further reduced by refining the calibration procedure, in particular by correcting the PSF of the device to reduce uncertainty related to aberrations in the optical system and pixel cross talk in the sensor.

## Figures and Tables

**Table 1:** Properties of the Raspberry Pi camera and custom optics.

Active Pixels	2592 × 1944 ≈ 5 Mpixel
Sensor	OmniVision OV5647
Pixel Dimension	1.4 µm × 1.4 µm
Sensor Size	3.67 mm × 2.74 mm
Digital resolution	10 bit = 1024 Digital Levels
Q.E. ^1^ @ 1 µm	2% < QE < 10%
FOV	9.96° × 7.47°
Line Readout Time	3.25 µs
Lens Focal Length	21.58 mm

^1^ Quantum Efficiency; the efficiency which incident photons generate a charge carrier.

**Table 2:** Components of combined uncertainty.

Calibration Uncertainties		
	SU (*k* = 1)	Sensitivity
Thermocouple calibration	0.4	1
Back wall discrepancy	0.5	1
Blackbody radiator $\varepsilon^{*}$	$0.02\cdot S_{meas}$	${\frac{\partial S_{model}}{\partial T}|}_{T}{}^{-1}$
Standard error of estimate	1.03	1
Camera use uncertainties		
Non-flat-field	$0.03\cdot S_{meas}$	${\frac{\partial S_{model}}{\partial T}|}_{T}{}^{-1}$
Noise	$B_{0}S_{meas}^{1/2}+B_{1}S_{meas}+B_{2}$	${\frac{\partial S_{model}}{\partial T}|}_{T}{}^{-1}$
$B_{0}\;=\;0.312289,\;B_{1}\;=\;0.005343,B_{2}\;=\;1.28808$		

**Table 3:** Measured Parameters.

$A_{0}\;$ ^1^	2 × 10^8^
$A_{1}\;$ ^1^	8.60591 × 10^−7^
$A_{2}\;$ ^1^	4.85862 × 10^−5^
Responsivity	9.646 × 10^16^ DL W^−1^
MFOV	1.35° (23.6 mrad)

^1^ Inferred from Sakuma-Hattori fit.
